# RPL35A promotes the progression of cholangiocarcinoma by mediating HSPA8 ubiquitination

**DOI:** 10.1186/s13062-024-00453-6

**Published:** 2024-02-23

**Authors:** Chengshuo Zhang, Yu Wang, Gang Wu, Ning Sun, Han Bai, Xuejian Li, Shuai Han, Haonan Zhou, Ruizhao Qi, Jialin Zhang

**Affiliations:** 1 Hepatobiliary Surgery Department, First Hospital of China Medical University, No.155, Nanjingbei street, 110001 Shenyang, Liaoning Province P. R. China; 2 Department of General Surgery, Anshan Central Hospital, No.51, South Zhonghua Road, Tiedong District, 114008 Anshan, Liaoning Province China; 3 Senior Department of General Surgery, the First Medical Center of Chinese PLA General Hospital, No.28, Fuxing Road, Haidian District, 100039 Beijing, China

**Keywords:** Cholangiocarcinoma, RPL35A, HSPA8, Ubiquitination

## Abstract

**Background:**

Cholangiocarcinoma (CCA) is a biliary epithelial malignant tumor with an increasing incidence worldwide. Therefore, further understanding of the molecular mechanisms of CCA progression is required to identify new therapeutic targets.

**Methods:**

The expression of RPL35A in CCA and para-carcinoma tissues was detected by immunohistochemical staining. IP-MS combined with Co-IP identified downstream proteins regulated by RPL35A. Western blot and Co-IP of CHX or MG-132 treated CCA cells were used to verify the regulation of HSPA8 protein by RPL35A. Cell experiments and subcutaneous tumorigenesis experiments in nude mice were performed to evaluate the effects of RPL35A and HSPA8 on the proliferation, apoptosis, cell cycle, migration of CCA cells and tumor growth in vivo.

**Results:**

RPL35A was significantly upregulated in CCA tissues and cells. RPL35A knockdown inhibited the proliferation and migration of HCCC-9810 and HUCCT1 cells, induced apoptosis, and arrested the cell cycle in G1 phase. HSPA8 was a downstream protein of RPL35A and overexpressed in CCA. RPL35A knockdown impaired HSPA8 protein stability and increased HSPA8 protein ubiquitination levels. RPL35A overexpression promoted CCA cell proliferation and migration. HSPA8 knockdown inhibited CCA cell proliferation and migration, and reversed the promoting effect of RPL35A. Furthermore, RPL35A promoted tumor growth in vivo. In contrast, HSPA8 knockdown suppressed tumor growth, while was able to restore the effects of RPL35A overexpression.

**Conclusion:**

RPL35A was upregulated in CCA tissues and promoted the progression of CCA by mediating HSPA8 ubiquitination.

**Supplementary Information:**

The online version contains supplementary material available at 10.1186/s13062-024-00453-6.

## Background

Cholangiocarcinoma (CCA) is a biliary epithelial malignant tumor and the second most common primary liver cancer [1]. It originates from different locations within the biliary tree and is divided into intrahepatic (iCCA), hepatic Peridoor (pCCA) or distal (dCCA) [2]. Risk factors for CCA include fibroinflammatory biliary disease (primary sclerosing cholangitis, intrahepatic bile duct stones, and liver fluke infection), cirrhosis, viral hepatitis, obesity-related liver disease, and diabetes. However, most CCAs have no identifiable cause [3, 4]. CCA is an aggressive and highly heterogeneous CCA with an increasing incidence worldwide over the past few decades, and currently accounts for approximately 15% and approximately 3% of all primary liver cancers. Gastrointestinal malignancies. Mortality accounts for approximately 2% of all cancer-related deaths worldwide each year, with overall CCA mortality higher in older patients than in younger patients, higher in men than in women, and higher in Asia than in Western regions [4, 5]. Because most patients with early-stage CCA are asymptomatic and the sensitivity of cytological and pathological diagnosis is limited, most patients present with advanced disease [6]. Currently, the only widely accepted curative treatment for iCCA is liver resection (LR). However, the probability of cure for LR is only 9.7%, and most iCCA patients also die within 3 years of diagnosis [7]. For patients with advanced or unresectable disease, local and systemic chemotherapy are the main treatment options with limited efficacy [8]. Therefore, a better understanding of the underlying molecular mechanisms of CCA pathogenesis and progression will help identify new treatment options.

As we all know, the ribosome is an organelle composed of a 40 S small subunit and a 60 S large subunit, and one of its outstanding features is to catalyze protein synthesis [9]. The human L35a ribosomal protein (RPL35A) gene is located on chromosome band 3q29-qter [10], encoding a ribosomal protein that is a component of the 60 S subunit [11]. ShRNA inhibition of RPL35A is essential for 28 S and 5.8 S rRNA maturation, 60 S subunit biogenesis, normal proliferation and cell survival [12]. It has been reported that a dominant mutation of the RPL35A gene on 3q29 is involved in the induction of Diamond-Blackfan anemia (DBA) disease [13]. B Xia et al. proposed that the RPL35A gene may be a potential pathogenic gene for osteoporosis [14]. In addition, the depletion of RPL35A significantly inhibited the growth of various cancer cell lines [15]. The interaction between rMgPa and RPL35 can promote the expression of proteins such as RPL35A, thereby promoting the proliferation of human urothelial cells [16]. However, the expression and role of RPL35A in CCA are still unclear.

In this study, we used immunohistochemical staining to find that RPL35A was highly expressed in CCA tissues and negatively correlated with the overall survival of CCA. In addition, knockdown of RPL35A significantly inhibited the proliferation and migration of CCA cells and promoted apoptosis, suggesting that RPL35A played an important role in the progression of CCA. We will further explore the regulatory mechanism of RPL35A in the progression of CCA, which will help to further study the development mechanism of CCA and help to identify new potential therapeutic targets for CCA.

## Methods

### Bioinformatics analysis

The Tumor Genome Atlas (TCGA) was used to analyze the differences in gene expression between cholangiocarcinoma and normal samples. The RNA-seq data of 36 CCA samples and 9 normal samples in TCGA were downloaded by GDC, and analyzed by R software. The data standardization was conducted by the method of estimating the dispersion in DEseq2. The differentially expressed genes were screened by DEseq2 and *P* value was adjusted by Benjamini-Hochberg. The screening criteria of differentially expressed genes were |fold change| ≥ 1.414 and *P* value < 0.05. The screening results were visualized by R software in the form of heat map. The mRNA expression of RPL35A and HSPA8 in CCA (CHOL) and normal samples was predicted by Gene Expression Profiling Interactive Analysis (GEPIA) online tool.

### Tissue chip and immunohistochemical staining

Tissue chip (HIBD-Ade100PG-01) was purchased from Shanghai Outdo Biotech Company (Shanghai, China), including 88 intrahepatic cholangiocarcinoma tumor tissues and 5 para-carcinoma tissues. All tissue-derived patients had signed patient informed consent. This experiment was approved by the Ethics committee of the First Hospital of China Medical University. Paraffin-embedded tissue sections were blocked with 3% H_2_O_2_ and 5% serum, respectively. After that, tissue sections were incubated with primary and secondary antibodies for 1 h at room temperature. After washing with 1 × PBST, the tissue sections were stained with DAB in the dark for 5 min, and then counterstained with hematoxylin for 15 s. Excess dye was washed away with running water, and tissue sections were dehydrated with absolute ethanol. Finally, the tissue sections sealed with neutral gum were observed under the microscope (Olympus) for staining results. The tissue staining results were evaluated by positive cell score × staining color intensity score, and the higher the score, the higher the protein expression. Information on primary and secondary antibodies used in immunohistochemical staining was provided in Supplementary Table 1.

### Cell culture

The 4 cholangiocarcinoma cell lines (HCCC-9810, HUCCT1, QBC939, and RBE cells) and human intrahepatic biliary epithelial cell line (HIBEC cells) used in this study were all from BeNa Culture Collection (China). HCCC-9810, HUCCT1 and HIBEC cells were grown in RPMI-1640 medium (Corning) containing 10% foetal bovine serum (FBS, Ausbian). QBC939 and RBE cells were grown in DMEM high glucose medium (Corning) containing 10% FBS. All cells were cultured at 37 °C in an incubator with 5% CO_2_.

### Construction of gene perturbation or overexpression cell models

Construction of knockdown slowing virus: Multiple RNA interference target sequences were designed using RPL35A or HSPA8 gene as a template (Supplementary Table 2). The synthesized single-stranded DNA oligo was annealed in a 90 °C water bath for 15 min to form a double-stranded DNA oligo. Subsequently, the double-stranded DNA oligo was ligated with the linearized vector, and the ligated product was transformed into E. coli competent cells for amplification. The ligation product was extracted by plasmid extraction technology, and then co-transfected into 293T cells with lentivirus carrying green fluorescent protein (GFP) and other helper plasmids. After 72 h, the cell supernatant was collected, and the lentivirus containing shRNA was extracted.

Construction of gene overexpression lentivirus: Using the RPL35A gene as a template, the corresponding forward and reverse primers were designed, and then the RPL35A gene fragment was amplified by PCR. The RPL35A gene fragment was exchanged with the linear vector, and then the exchanged product was transformed into E. coli competent cells for amplification. The ligation product was extracted by plasmid extraction technology, and then co-transfected into 293T cells with GFP-carrying lentivirus and other helper plasmids. After 72 h, the cell supernatant was collected, and the lentivirus containing the RPL35A gene fragment was extracted.

Construction of gene knockdown or overexpression cholangiocarcinoma cells: HCCC-9810 and HUCCT1 cells (2 × 10^5^ cells) were infected with lentiviruses containing shRNA or RPL35A gene fragments at a titer of 1 × 10^8^ TU/mL. After 20 h, the medium was replaced with a new one. The cells were further cultured for 72 h, and then the infection efficiency was assessed by observing fluorescence under a microscope.

### Real-time quantitative PCR (RT-qPCR)

Trizol (Sigma) was used to extract total RNA from HCCC-9810 and HUCCT1 cells, and then Nanodrop 100 spectrophotometer (Thermo) was used to analyze the concentration and quality of the extracted RNA. RNA was reverse transcribed to cDNA using Hiscript QRT supermix for qPCR (+ gDNA WIPER) (Vazyme) according to the manufacturer’s instructions. The cDNA, SYBR Green mastermixs (Vazyme), forward and reverse primers, and other materials were prepared in proportion to prepare the reaction system, and then Real-time PCR was performed on a Real time PCR instrument (ABI) using a two-step method, and the melting curve was produced. The relative expression levels of genes were then calculated using 2-^ΔΔCt^. The relevant primers used in the study are as follows:

RPL35A: forward primers, 5’- GAAGTGTTTACGCCCGAGAT − 3’, reverse primers, 5’- CGAGTTACTTTTCCCCAGATGAC − 3’; GAPDH: forward primers, 5’- TGACTTCAACAGCGACACCCA − 3’, reverse primers, 5’- CACCCTGTTGCTGTAGCCAAA − 3’.

### Western blot (WB)

HCCC-9810 and HUCCT1 cells were lysed and total protein was extracted. BCA Protein Assay Kit (HyClone-Pierce) was used to detect protein concentration. Then 20 µg protein was used for SDS-PAGE. The proteins were then transferred to PVDF membranes, which were then blocked with 1 × TBST solution containing 5% skim milk for 1 h at room temperature. PVDF membranes were incubated with primary and secondary antibodies for 1 h at room temperature, and then washed three times with 1 × TBST for 10 min each time. Immobilon Western Chemiluminescent HRP Substrote Kit (Millipore) was used to develop protein color, and then chemiluminescence was performed with a chemiluminescence imager (GE), and the protein bands were photographed. Information on primary and secondary antibodies used in western blot assay was provided in Supplementary Table 1.

### Celigo cell count assay

The HCCC-9810 and HUCCT1 cell suspensions of each group after infection with lentivirus were added to a 96-well plate (100 µL/well) at a density of 1000 cells per well. After 24 h, Celigo (Nexcelom) was used to detect the same field at the same time for 5 consecutive days to obtain the scanning images, and the cells were counted. Cell growth curves were drawn based on cell count results and time points.

### Flow cytometry

Apoptosis detection by flow cytometry: When HCCC-9810 and HUCCT1 cells were grown to 85% coverage, they were harvested after trypsinization. Cells were washed once with pre-chilled D-Hanks and 1 × binding buffer, respectively. The cells were resuspended in 200 µL of 1 × binding buffer, and then incubated with 10 µL of Annexin V-APC (apoptosis kit, eBioscience) for 15 min at room temperature in the dark. Finally, the level of apoptosis was detected by flow cytometry (Millipore).

Cell cycle detection by flow cytometry: When the coverage of HCCC-9810 and HUCCT1 cells exceeded 70%, cells were harvested after trypsinization. After washing once with pre-cooled PBS, cells were fixed in 70% ethanol pre-cooled at 4 °C for 1 h. After washing with PBS, the cells were resuspended with the prepared staining solution, and the cell cycle was detected by flow cytometry. Preparation ratio of cell staining solution: 40 × PI solution (2 mg/ml, Sigma): 100 × RNase solution (10 mg/ml, TakaRa): 1 × PBS = 25:10:1000.

### Transwell assay

100 µL serum-free was added to the upper chamber of Transwell (Corning), which was placed in the incubator for 1 h. 600 µL medium with 30% FBS was added to the lower chamber. After removing the serum-free medium in the upper chamber, 100 µL HCCC-9810 or HUCCT1 cell suspension (50,000 cells/well) was then added. The upper chamber was transferred to the lower chamber, and incubated in the incubator for 72 h. The medium in the upper chamber was removed and non-metastatic cells were gently removed with a cotton swab. The upper chamber was transferred to a new lower chamber with 400 µL staining solution. The upper chamber was washed 5 min later, and the transferred cells were photographed and counted with a microscope.

### Wound healing

100 µL HCCC-9810 or HUCCT1 cell suspension was added to a 96-well plate (50,000 cells/well). After 24 h, the medium was replaced with low-concentration FBS (0.5%) medium, and then a 96 Wounding Replicator (VP scientific) was used to nudge the lower central part of the well to form a scratch. After washing with FBS-free medium, medium containing 0.5% FBS was added and scanned with Cellomics (Thermo), which was recorded as 0 h. Cells were cultured at 37 °C in an incubator with 5% CO_2_. 96-well plates were scanned at 8 and 24 h. Cellomics was employed to analyze the scanned images to assess the ability of cells to migrate.

### Immunoprecipitation-mass spectrometry (IP-MS)

HCCC-9810 cells in RPL35A overexpression group or control group were lysed, and then total protein was extracted. Proteins were isolated in 10% SDS-PAGE gels and stained with Coomassie brilliant blue. Differentially expressed protein bands were excised from SDS-PAGE gels and digested with Trypsin (Promega) for 20 h at 37 °C. After the chromatographic column was equilibrated with 95% A solution (0.1% formic acid aqueous solution) and 5% B solution (0.1% formic acid in acetonitrile aqueous solution), the sample was loaded onto the Trap column from the autosampler column. The mass spectrometer Exactive (Thermo Fisher) was applied to detect the sample, and Easy-nLC 1000 (Thermo Fisher) was used to analyze the sample. Finally, Proteome Discoverer1.4 software was used to retrieve the corresponding database, and the mass spectrometry data were analyzed to obtain the qualitative identification information of the target protein.

### Co-immunoprecipitation (Co-IP)

Total protein was extracted after lysing HCCC-9810 and HUCCT1 cells. 1.0 mg protein was taken out and incubated with reference antibody overnight at 4 °C with inversion. Protein-antibody complexes were incubated with 200 µL beads for 1 h at 4 °C with inversion. The protein-antibody-beads complex was washed twice with IP lysis buffer, and then subjected to SDS-PAGE electrophoresis. Afterwards, the same operation as the above western blot assay was performed to detect the expression of related proteins. Information on primary and secondary antibodies used in Co-IP assay was provided in Supplementary Table 1.

### Protein stability assay

To evaluate the effect of RPL35A on HSPA8 protein stability, HCCC-9810 and HUCCT1 cells were treated with the protein synthesis inhibitor cycloheximide (CHX). Cells were harvested at 0, 2, 4 and 8 h after CHX treatment, and the protein was extracted. The HSPA8 protein levels were detected by western blot. Information on primary and secondary antibodies used in this assay was provided in Supplementary Table 1.

### Ubiquitination level detection assay

HCCC-9810 and HUCCT1 cells were treated with proteasome inhibitor MG-132. After 24 h, the protein in the cells was extracted. 20 µg protein was removed, and the level of HSPA8 protein was detected by western blot. Another 1.0 mg protein was taken out and incubated with the reference antibody overnight at 4 °C with inversion, and then incubated with 200 µL beads for 1 h at 4 °C with inversion. Finally, the ubiquitin level of HSPA8 protein was detected by Co-IP using anti-Ubiquitin antibody. Information on primary and secondary antibodies used in this assay was provided in Supplementary Table 1.

### Nude mouse tumor model

4-week-old female BALB/c nude mice were purchased from Jiangsu Jicui Yaokang Biotechnology Co., Ltd. All animal-related experiments were performed with the approval of the Institutional Animal Care and Use Committee of China Medical University. 1.0 × 10^7^ HUCCT1 cells were injected subcutaneously into nude mice. From the 7th day of cell injection, the long and short diameters of the tumors in nude mice were measured every 5 days, and the tumor volume was calculated by formula to draw the tumor growth curve. Tumor volume = π/6 × L × W × W, i.e. = 3.14/6 × L × W × W. L represented the long diameter and W represented the short diameter. After 27 days, nude mice were euthanized, tumors were removed and weights were measured. Afterwards, the expression of Ki67 protein in different groups of tumor tissues was detected by immunohistochemical staining. Details of the antibodies were provided in Supplementary Table 1.

### Statistical analysis

Cell and animal experimental data in this study were expressed as mean ± standard deviation (SD). SPSS software was used for statistical analysis, and GraphPad Prism software was used to draw statistical graphs. The results of immunohistochemical staining to detect the difference of RPL35A expression in CCA and para-carcinoma tissues were analyzed by Sign test. Mann-Whitney U was employed to assess whether the expression of RPL35A in CCA tissue was significantly different in pathological data. Spearman correlation analysis was used to evaluate the correlation between the expression of RPL35A and pathological data. The association of RPL35A expression with overall survival in patients with CCA was assessed by Rank analysis in Kaplan-Meier survival analysis. The t-test was used for statistical analysis between two groups, and statistical analysis of three or more groups was performed by one-way ANOVA. When *P* was less than 0.05, the difference was considered statistically significant.

## Results

### RPL35A was upregulated in CCA and RPL35A knockdown inhibited CCA cell proliferation and migration

**Fig. 1:**
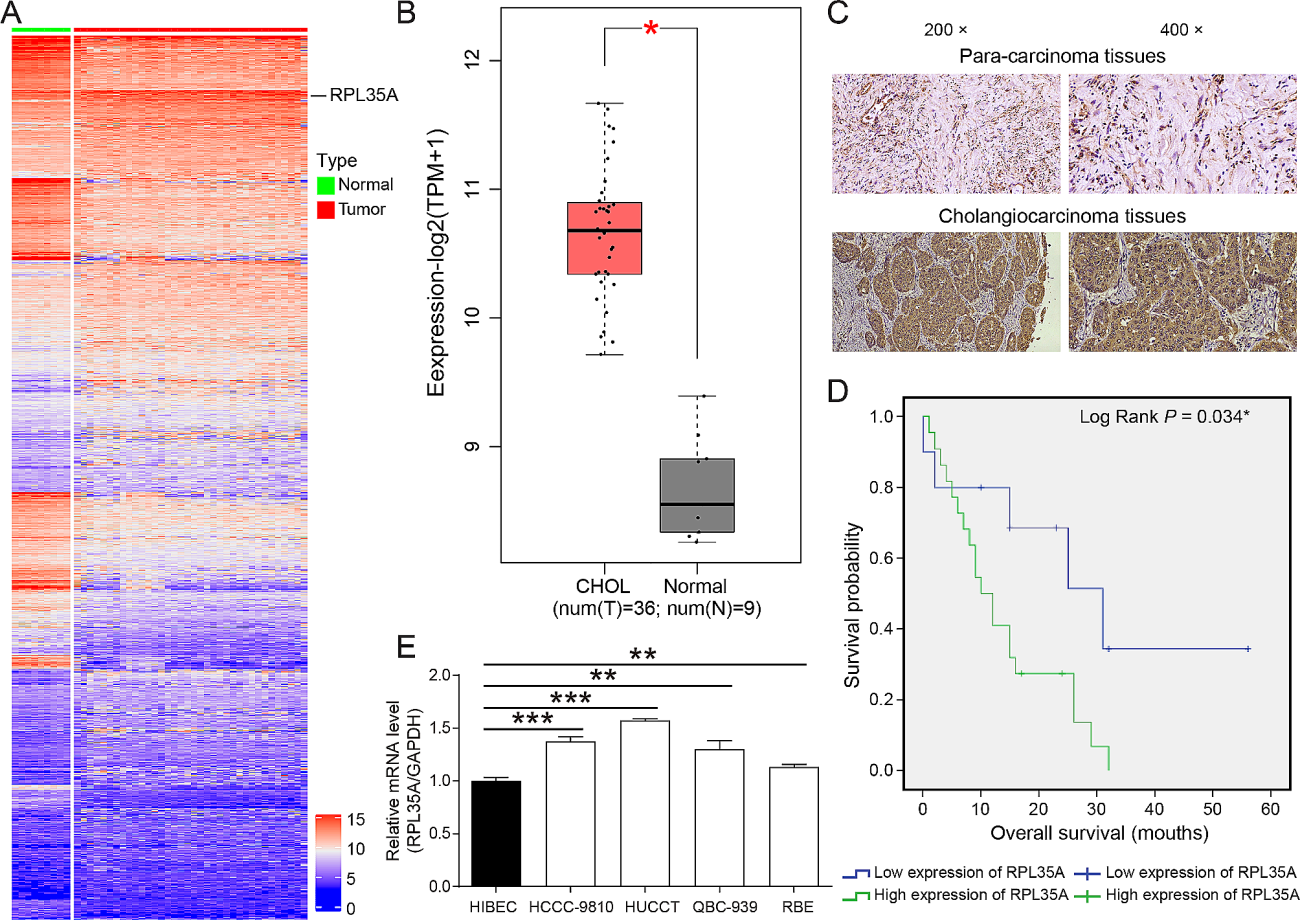
RPL35A was upregulated in CCA **(A)** The heat map displayed the significantly differentially expressed genes in CCA. The data were obtained from TCGA database and analyzed by R software. **(B)** The RPL351 levels in CCA and normal samples was predicted by GEPIA online tool. **(C)** Immunohistochemical staining was used to evaluate the expression of RPL35A protein in normal bile duct tissues and CCA tissues. **(D)** Kaplan-Meier survival analysis shows that high expression of RPL35A has poorer survival. **(E)** RPL35A expression in 4 CCA cell lines (HCCC-9180, HUCCT1, QBC-939, and RBE) and human intrahepatic biliary epithelial cell line (HIBEC cells) was detected by RT-qPCR. *P < 0.05, **P < 0.01, ***P < 0.001

There were 36 CCA samples and 9 normal samples in TCGA database, which were used to screen differentially expressed genes in CCA samples. The results were visualized in the heat map by R software, and RPL35A was one of the significantly upregulated genes (Fig. 1A). The mRNA levels of RPL35A in CCA and normal samples were predicted by GEPIA online tool, and compared with normal samples, RPL35A was markedly higher expressed in CCA samples (Fig. 1B). Through the immunohistochemical staining analysis of 88 cases of CCA tissues and 5 cases of para-carcinoma tissues, the expression of RPL35A protein in CCA tissues was demonstrated to be higher than that in para-carcinoma tissues (Fig. 1C **and** Table 1). Additionally, RPL35A had a higher level in CCA cell lines compared to HIBEC cells (Fig. 1E). Further statistical analysis showed that the expression of RPL35A was significantly different in patient gender, and was higher in male patients (Tables 2 and 3). Besides, Kaplan-Meier survival analysis revealed that the expression of RPL35A was significantly correlated with the overall survival (OS) of CCA, that is, as the expression of RPL35A gene was increased, the survival period was shortened (Fig. 1D). These results suggested that RPL35A played a crucial role in the progression and prognosis of CCA.

**Table 1:** Expression pattern of RPL35A in cholangiocarcinoma cancer tissues and para-carcinoma tissues revealed in immunohistochemistry analysis

RPL35A expression	Tumor tissue		Para-carcinoma tissue		***P*** value
	Cases	Percentage	Cases	Percentage	
Low	37	42.0%	5	100.0%	< 0.001***
High	51	58.0%	0	0%	

**Table 2:** Relationship between RPL35A expression and tumor characteristics in patients with cholangiocarcinoma cancer

Features	No. of patients	RPL35A expression		***P*** value
		low	high	
All patients	88	37	51	
Age (years)				0.080
< 61	43	14	29	
≥ 61	45	23	22	
Gender				0.042*
Male	56	19	37	
Female	32	18	14	
Grade				0.693
I	1	1	0	
II	49	19	30	
III	37	16	21	
IV	1	1	0	
Tumor size				0.830
< 6.5 cm	44	18	26	
≥ 6.5 cm	44	19	25	
Metastasis				0.743
M0	84	35	49	
M1	4	2	2	

**Table 3:** Relationship between RPL35A expression and tumor characteristics in patients with cholangiocarcinoma cancer

		RPL35A
Gender	Spearman correlation	-0.218*
	Significance(two-tails)	0.042*
	N	88

**Fig. 2:**
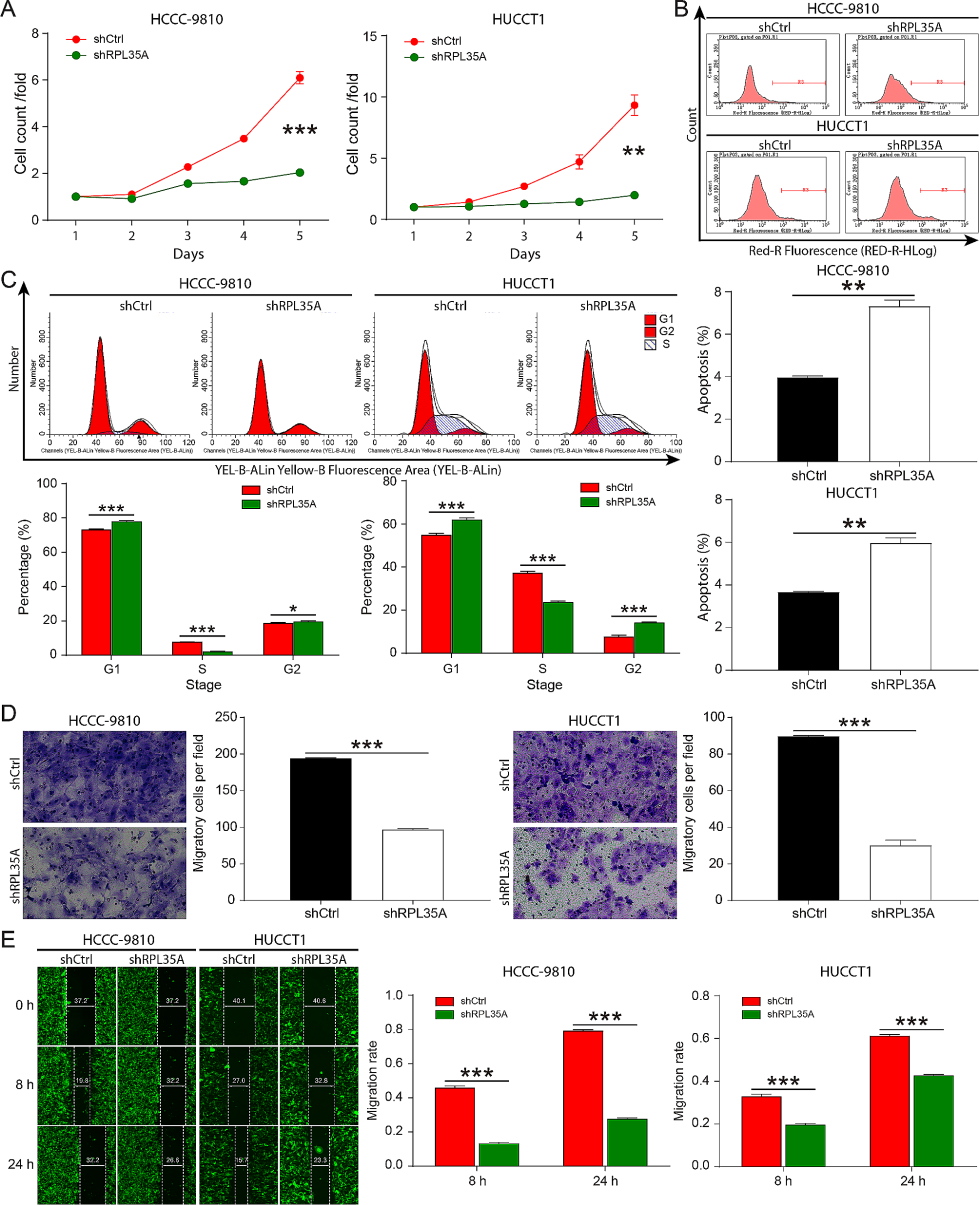
Knockdown of RPL35A inhibited CCA cell proliferation and migration **(A)** The proliferation of HCCC-9180 and HUCCT1 cells after RPL35A knockdown was detected by Celigo cell count assay. **(B)** The results of flow cytometry showed increased apoptosis in HCCC-9180 and HUCCT1 cells after RPL35A knockdown. **(C)** Flow cytometry was used to assess the role of RPL35A in the cycle progression of HCCC-9180 and HUCCT1 cells. **(D)** Transwell assay was performed to evaluate the effect of RPL35A knockdown on the migration of HCCC-9180 and HUCCT1 cells. **(E)** Wound healing assay was applied to verify the effect of RPL35A knockdown on the migration of HCCC-9180 and HUCCT1 cells. *P < 0.05, **P < 0.01, ***P < 0.001

To investigate the role of RPL35A on the progression of CCA, the expression of RPL35A was examined by RT-qPCR in four CCA cells (HCCC-9180, HUCCT1, QBC-939, and RBE) and human intrahepatic biliary epithelial cell line (HIBEC cells), which indicated that RPL35A was wide overexpressed in CCA cell lines (Fig. 1E). Three shRNAs targeting RPL35A were designed to infect HCCC-9810 cells respectively, and found that shRPL35A-1 had a more obvious inhibitory effect on the expression of RPL35A and was used for follow-up experimental exploration (Fig. S1A). RT-qPCR and western blot results showed that when HCCC-9810 and HUCCT1 cells were infected with shRPL35A, both the mRNA and protein levels of RPL35A were significantly reduced, suggesting that RPL35A knockdown HCCC-9810 and HUCCT1 cells were successfully constructed (Fig.S1B-S1C). RPL35A knockdown not only slowed the proliferation of HCCC-9810 and HUCCT1 cells (Fig. 2A and Fig. S2A-S2B), but also induced cell apoptosis (Fig. 2B). Furthermore, downregulation of RPL35A reduced the proportion of S phase cells (Fig. 2C), and inhibited cell migration (Fig. 2D and E).

### RPL35A upregulated HSPA8 protein level through inhibiting ubiquitination modification

**Fig. 3:**
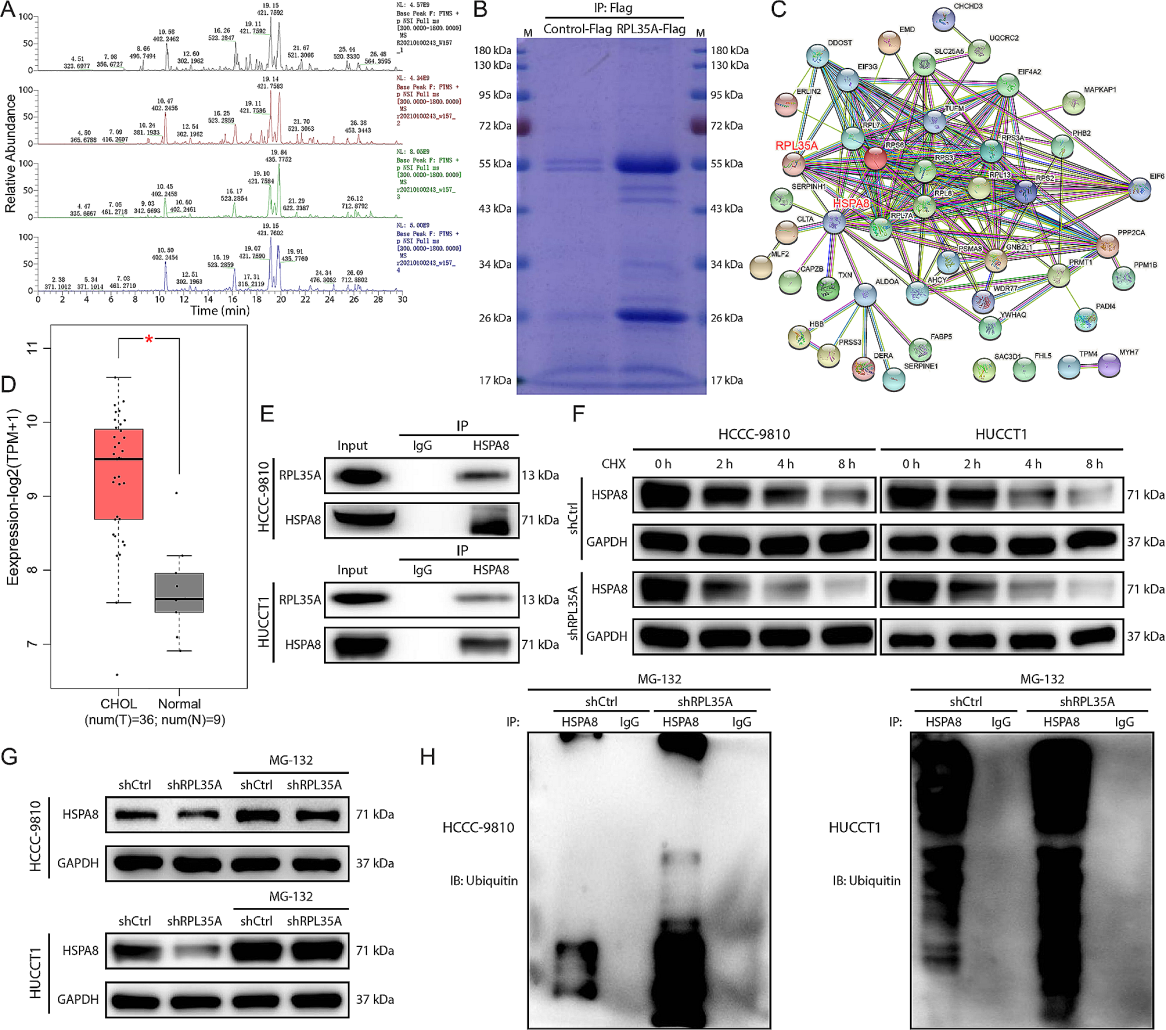
RPL35A knockdown promoted the ubiquitination of HSPA8 protein **(A)** Base peak graph of IP-MS detection results. **(B)** Coomassie brilliant blue staining was employed to detect protein expression in the RPL35A overexpression group and the control group and the protein bands was identified by mass spectrometry. **(C)** The protein-protein interaction network diagram showed the results of predicting proteins that might interact with RPL35A using STRING online tool. **(D)** The HSPA8 levels in CCA and normal samples was predicted by GEPIA online tool. **(E)** The interaction between RPL35A and HSPA8 was determined by Co-IP. **(F)** HCCC-9810 and HUCCT1 cells in RPL35A knockdown group and control group were treated with CHX, and the HSPA8 protein levels were detected by western blot at 0, 2, 4, and 8 h, respectively. **(G)** HSPA8 protein levels in HCCC-9810 and HUCCT1 cells treated with/without MG-132 in RPL35A knockdown group and control group were detected by western blot. **(H)** Co-IP was used to detect the ubiquitin level of HSPA8 protein after MG-132 treatment of HCCC-9810 and HUCCT1 cells. *P < 0.05

To further explore the molecular mechanism of RPL35A in CCA progression, we screened proteins that might interact with RPL35A by IP-MS (Fig. 3A). Coomassie brilliant blue staining showed the protein expression in the RPL35A overexpression group and the control group, which indicated that there were some significantly differentially expressed protein bands, including HSPA8 (Fig. 3B). The STRING online tool was used to predict the proteins that might interacted with RPL35A and was showed in the protein-protein interaction (PPI) network, which revealed the interaction between RPL35A and HSPA8 (Fig. 3C). According to the predicting results of GEPIA online tool, HSPA8 was markedly higher expressed in CCA samples compared with normal samples (Fig. 3D), and the Co-IP results further verified that RPL35A directly acted on HSPA8 (Fig. 3E).

It is well known that ubiquitination is an important post-translational modification (PTM) mediated by ubiquitin-activating enzymes (E1), ubiquitin-conjugating enzymes (E2) and ubiquitin ligases (E3). The ubiquitin proteasome system (UPS) regulates a wide range of cellular processes by directing protein degradation [17]. Given that RPL35A directly interacted with HSPA8 at the protein level, we speculated whether RPL35A affected HSPA8 protein levels by regulating ubiquitination modification. HCCC-9810 cells in RPL35A knockdown group and shCtrl group were treated with the protein synthesis inhibitor CHX, respectively, and RPL35A knockdown significantly accelerated the reduction in HSPA8 protein levels, suggesting that RPL35A downregulation weakened HSPA8 protein stability (Fig. 3F). The proteasome inhibitor MG-132 reversed the downregulation of HSPA8 protein by RPL35A knockdown (Fig. 2G). Furthermore, when RPL35A was knocked down, the ubiquitin level of HSPA8 protein was significantly upregulated (Fig. 3H). The above results revealed that RPL35A knockdown decreased HSPA8 protein level by upregulating HSPA8 ubiquitination modification.

### RPL35A promoted CCA progression in vitro and in vivo by upregulating HSPA8

We then investigated whether the effect of RPL35A on CCA progression was mediated by HSPA8. The levels of HSPA8 in HCCC-9180, HUCCT1, QBC-939 and RBE cells was obviously higher than that in HIBEC cells (Fig. S3A). Three shHSPA8s were infected HCCC-9810 cells, respectively, and found that shHSPA8-1 had a significant inhibitory effect on the expression of HSPA8 and was used for follow-up experimental exploration (Fig. S3B). RT-qPCR and western blot results showed that RPL35A overexpression in HCCC-9810 and HUCCT1 cells increased the expression of HSPA8. The expression of HSPA8 in HCCC-9810 and HUCCT1 cells infected with shHSPA8 lentivirus was decreased, which was reversed by overexpressing RPL35A (Fig. S3C-S3D).

**Fig. 4:**
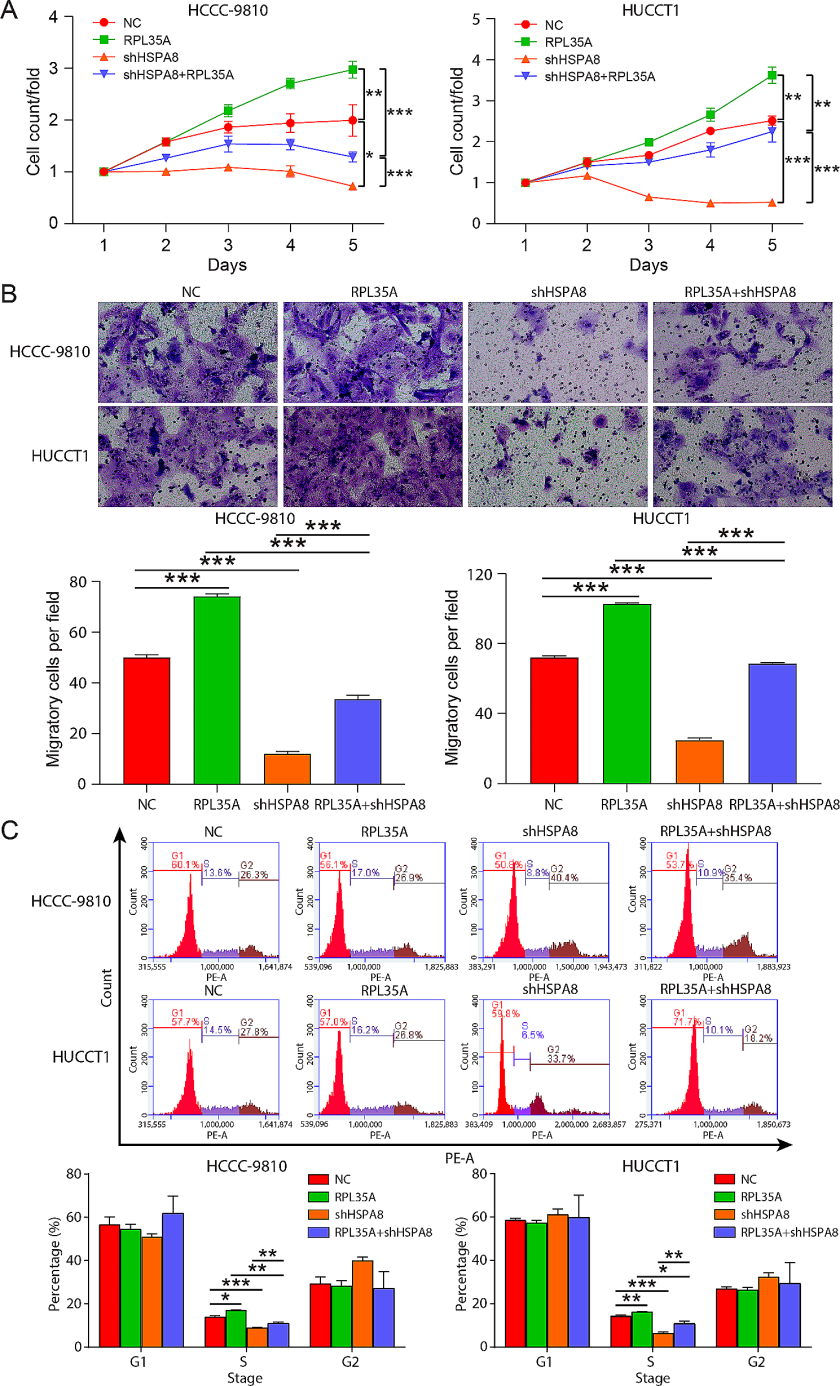
RPL35A promoted CCA cell proliferation and migration by upregulating HSPA8 **(A)** Celigo cell count assay was performed to detect the proliferation of HCCC-9180 and HUCCT1 cells in each group. **(B)** Transwell assay was used to evaluate the migration of HCCC-9180 and HUCCT1 cells in each group. **(C)** The cell cycle of HCCC-9810 and HUCCT1 cells was assessed by flow cytometry. *P < 0.05, **P < 0.01, ***P < 0.001

**Fig. 5:**
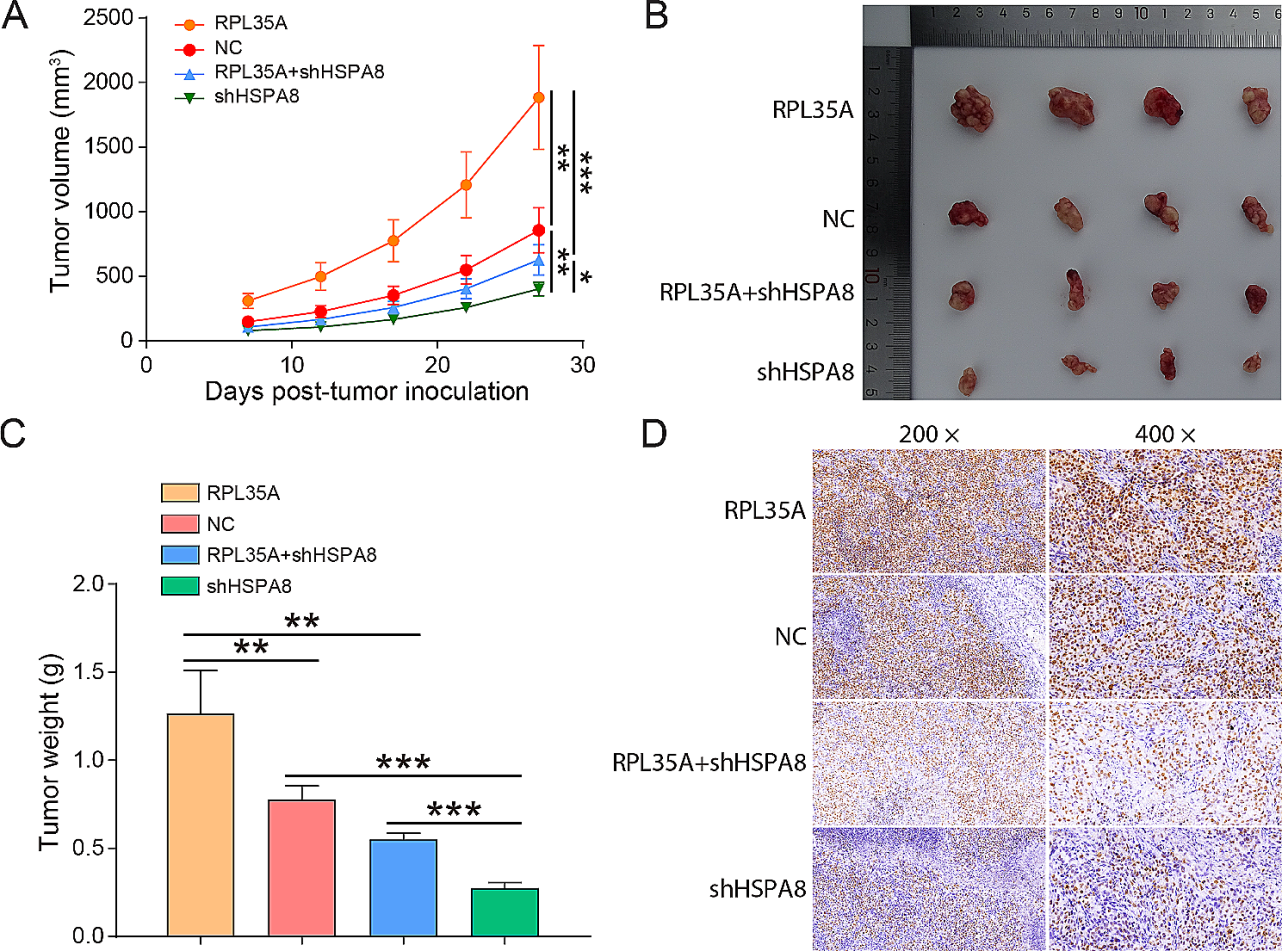
RPL35A promoted tumor growth in vivo by upregulating HSPA8 **(A)** Nude mice were injected subcutaneously with HCCC-9810 cells in each group, and the tumor growth was detected at different time points. **(B)** Solid tumors were removed from nude mice after nude mice were sacrificed. **(C)** The weight of tumors. **(D)** The level of Ki-67 protein in tumor tissues were assessed by immunohistochemical staining. *P < 0.05, **P < 0.01, ***P < 0.001

RPL35A overexpression significantly enhanced the proliferation and migration abilities of HCCC-9810 and HUCCT1 cells (Fig. 4A and B and Fig. S4A-S4B). In contrast, HSPA8 knockdown had the opposite effect, significantly attenuating the proliferation and migration of CCA. What’s more, HSPA8 downregulation reversed the effects of RPL35A overexpression on the proliferation and migration of CCA (Fig. 4A and B and Fig. S4A-S4B). Additionally, flow cytometry results indicated an increased S phase of HCCC-9810 and HUCCT1 cells in the RPL35A overexpression group and a declined percentage of S phase in the HSPA8 knockdown group, while HSPA8 restrained the increase caused by RPL35A upregulation (Fig. 4C). Further in vivo experiments suggested that RPL35A overexpression significantly accelerated tumor growth in vivo, whereas HSPA8 knockdown significantly slowed tumor growth. HSPA8 knockdown also reversed the promoting effect of RPL35A overexpression (Fig. 5A and C). Immunohistochemical staining detected the protein level of Ki67 in tumor tissues, and found overexpression of RPL35A upregulated Ki67 protein, and knockdown of HSPA8 downregulated Ki67 protein, which was consistent with the change trend of cell proliferation in vitro (Fig. 5D).

## Discussion

CCA is a highly lethal hepatobiliary tumor with an increasing incidence [18]. As an aggressive malignancy, CCA is characterized by difficult early diagnosis, limited treatment options, poor prognosis and high mortality [19]. Therefore, there is an urgent need to deeply explore the regulatory mechanisms of CCA progression to develop more effective treatments. A previous study showed that RPL35A was significantly upregulated in gastric cancer and positively correlated with tumor invasion [20]. In addition, RPL35A was highly expressed in hepatocellular carcinoma, and was associated with poor prognosis [21]. These were suggested that RPL35A played a tumor-promoting role in tumor progression, but its role in CCA was unclear. The expression of RPL35A in CCA and normal samples was predicted by R software and GEPIA online tool, and found that RPL35A upregulated in CCA samples. Besides, the expression of RPL35A in CCA and para-carcinoma tissues was determined by immunohistochemical staining, which indicated that the expression of RPL35A was significantly higher in CCA, and was negatively correlated with the overall survival of patients with CCA. Downregulation of RPL35A significantly inhibited gastric cancer cell proliferation and migration, enhanced apoptosis and arrested cell cycle [20]. To verify the effect of RPL35A on the function of CCA cells, RPL35A knockdown HCCC-9810 and HUCCT1 cell models were constructed. Knockdown of RPL35A inhibited the proliferation, migration and enhanced apoptosis of CCA cells. Similar to the findings of Reyes Babiano et al. [22], RPL35A knockdown also delayed the G1 phase of the CCA cell cycle and reduced the S phase. Furthermore, RPL35A overexpression promoted the proliferation and migration of HCCC-9810 and HUCCT1 cells. The above results demonstrated the promoting effect of RPL35A on the proliferation and migration of CCA cells in vitro. However, the specific molecular mechanism of RPL35A’s role needs to be further explored.

Previous studies showed that the 60 S ribosomal subunit protein played a key role in mRNA translation and protein synthesis. As a component of the 60 S ribosomal subunit, RPL35A might also regulate the expression of downstream proteins through interacting with them. By performing IP-MS on the proteins in HCCC-9810 cells in the RPL35A overexpression group and control group, combined with the PPI network, we found that HSPA8 was a downstream protein regulated by RPL35A. HSPA8 was upregulated in CCA samples and CAA cell lines. Co-IP results confirmed the interaction between RPL35A and HSPA8. These results suggested that HSPA8 was a downstream protein of RPL35A. Heat shock 70 kDa protein 8 (HSPA8), also known as heat shock homologous protein 70 (HSC70), belongs to the heat shock protein 70 (HSP70) family and is a constitutively expressed molecular chaperone [23]. HSPA8 was located in the cytosol, nucleus, and extracellular exosomes [24], and played an important physiological role in protein folding and degradation [25]. Eukaryotic cells operated two major protein degradation systems, the ubiquitin-proteasome system (UPS) and autophagy [26]. Previous studies indicated that the E3 ubiquitin ligase CHIP functioned together with stress-induced HSP70 and constitutive HSC70 chaperones to participate in protein sorting, as a co-chaperone for refolding and for catalyzing the ubiquitination of substrates. When the chaperone proteins HSP70 and HSC70 did not carry client proteins, CHIP catalyzed their polyubiquitination and subsequent proteasomal degradation [27]. Our results showed that the rate of HSPA8 protein decline was significantly increased after CHX treatment of RPL35A knockdown cells. MG-132 reversed the downregulation of HSPA8 protein by RPL35A knockdown. Furthermore, downregulation of RPL35A also enhanced HSPA8 protein ubiquitination. All of these revealed that RPL35A enhanced HSPA8 stability by inhibiting ubiquitination modification.

HSPA8 was found to be overexpressed in various cancer cells, which was essential for the growth of cancer cells [28–30]. Furthermore, depletion of HSPA8 inhibited cell growth, induced apoptosis and arrested cell cycle in solid human tumors [31, 32]. Our study found that HSPA8 knockdown inhibited the proliferation and migration of HCCC-9810 and HUCCT1 cells, and decreased the percentage of S phase cells, besides, reversed the effects of RPL35A overexpression on CCA cells. In vivo, HSPA8 downregulation also inhibited tumor growth and decreased Ki-67 protein expression in tumor tissues, which was opposite to the effect of RPL35A overexpression on tumor growth. The above results proved that RPL35A promoted the progression of CCA by acting on HSPA8 in vitro and in vivo. However, this study still has some shortcomings. We should expand the number of clinical samples and verify the correlation of RPL35A and HSPA8 expression in CCA tissues. In addition, studies showed that HSPA8 was a key molecular regulator of chaperone-mediated autophagy [33], we should further explore whether RPL35A regulates autophagy through HSPA8 and thus plays a tumor-promoting role in the progression of CCA.

## Conclusion

Taken together, RPL35A was upregulated in CCA and promoted the proliferation and migration of CCA cells and tumor growth. In addition, RPL35A was able to upregulate the expression of HSPA8 protein by mediating ubiquitination. This study not only clarified the role of RPL35A in the progression of CCA, but also preliminarily explored the molecular mechanism of RPL35A promoting CCA, suggesting that RPL35A might be a new potential therapeutic target for CCA, which was helpful for the development of new therapeutic strategies.

### Electronic supplementary material

Below is the link to the electronic supplementary material.

Supplementary Material 1

## Data Availability

The data used and analyzed during the current study are available from the corresponding author on reasonable request.
