# Supplementary material for: RPL35A promotes the progression of cholangiocarcinoma by mediating HSPA8 ubiquitination
Source: Biol Direct. 2024 Feb 23;19:16. doi: 10.1186/s13062-024-00453-6 (PMC10885515; doi:10.1186/s13062-024-00453-6)
Supplement: Supplementary file 1 — Supplementary Material 1 [file 13062_2024_453_MOESM1_ESM.docx]

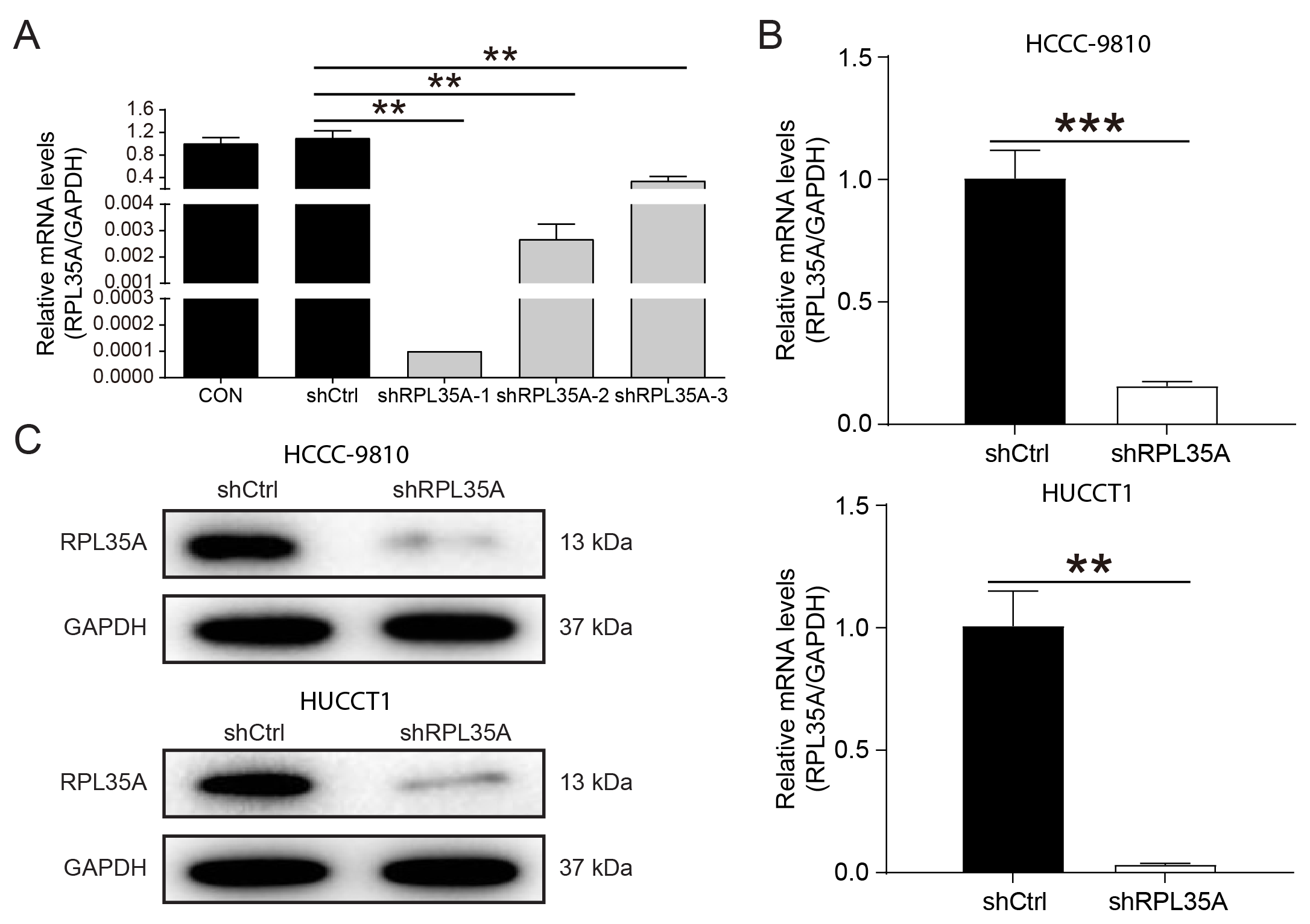


**Supplementary figure 1** **Construction of RPL35A knockdown CCA cells**

A. RT-qPCR was used to detect the levels of RPL35A mRNA in HCCC-9180 cells infected with three shRPL35A lentiviruses. B. The expression of RPL35A mRNA in HCCC-9180 and HUCCT1 cells infected with shRPL35A lentivirus was detected by RT-qPCR. C. The expression of RPL35A protein in HCCC-9180 and HUCCT1 cells infected with shRPL35A lentivirus was detected by western blot. ***P* < 0.01, ****P* < 0.001.


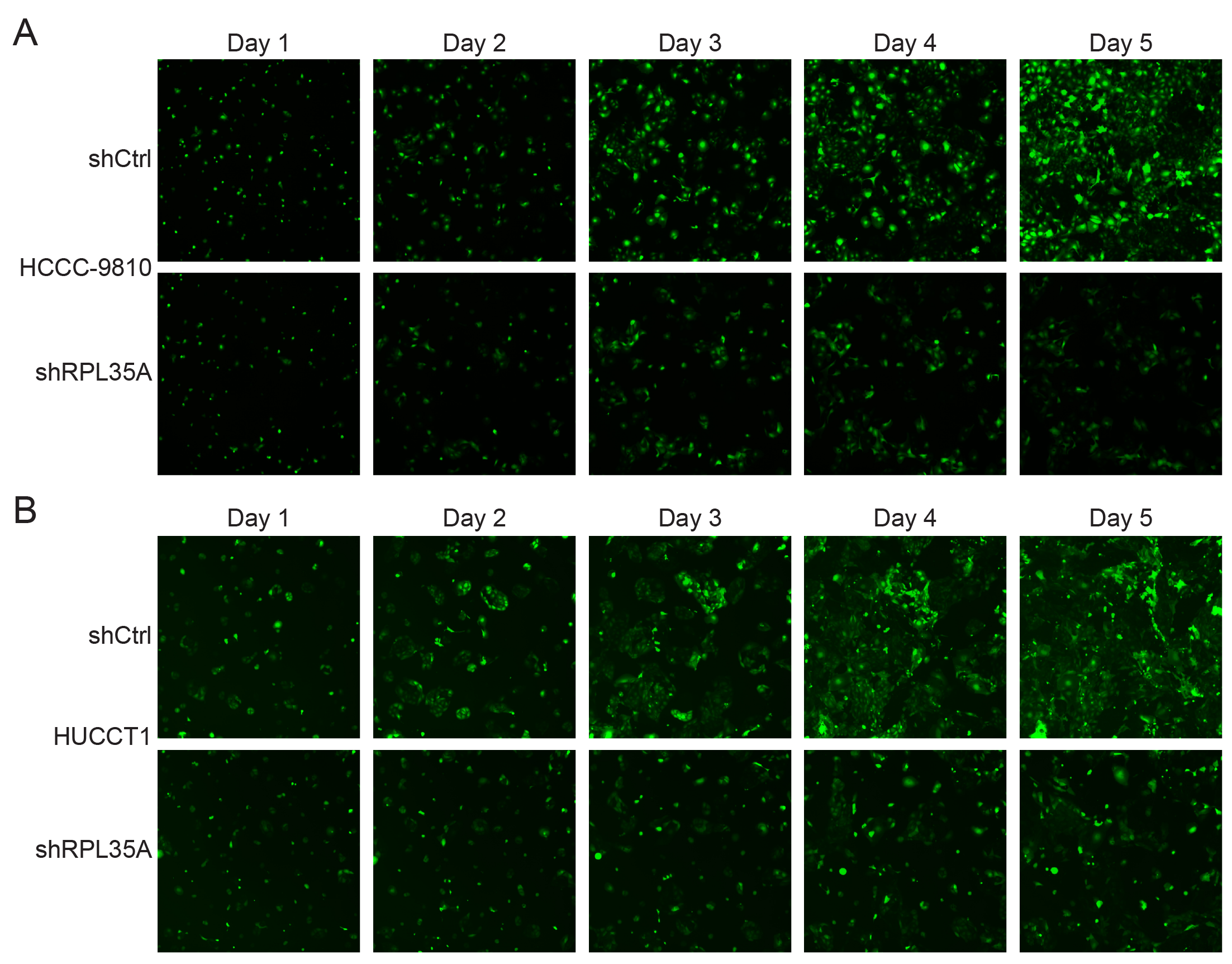


**Supplementary figure 2 Representative images of CCA cell proliferation assessed by Celigo cell count assay**

A. Representative images of the proliferation of HCCC-9180 cells after RPL35A knockdown detected by Celigo cell count assay. B. Representative images of the proliferation of HUCCT1 cells after RPL35A knockdown detected by Celigo cell count assay.


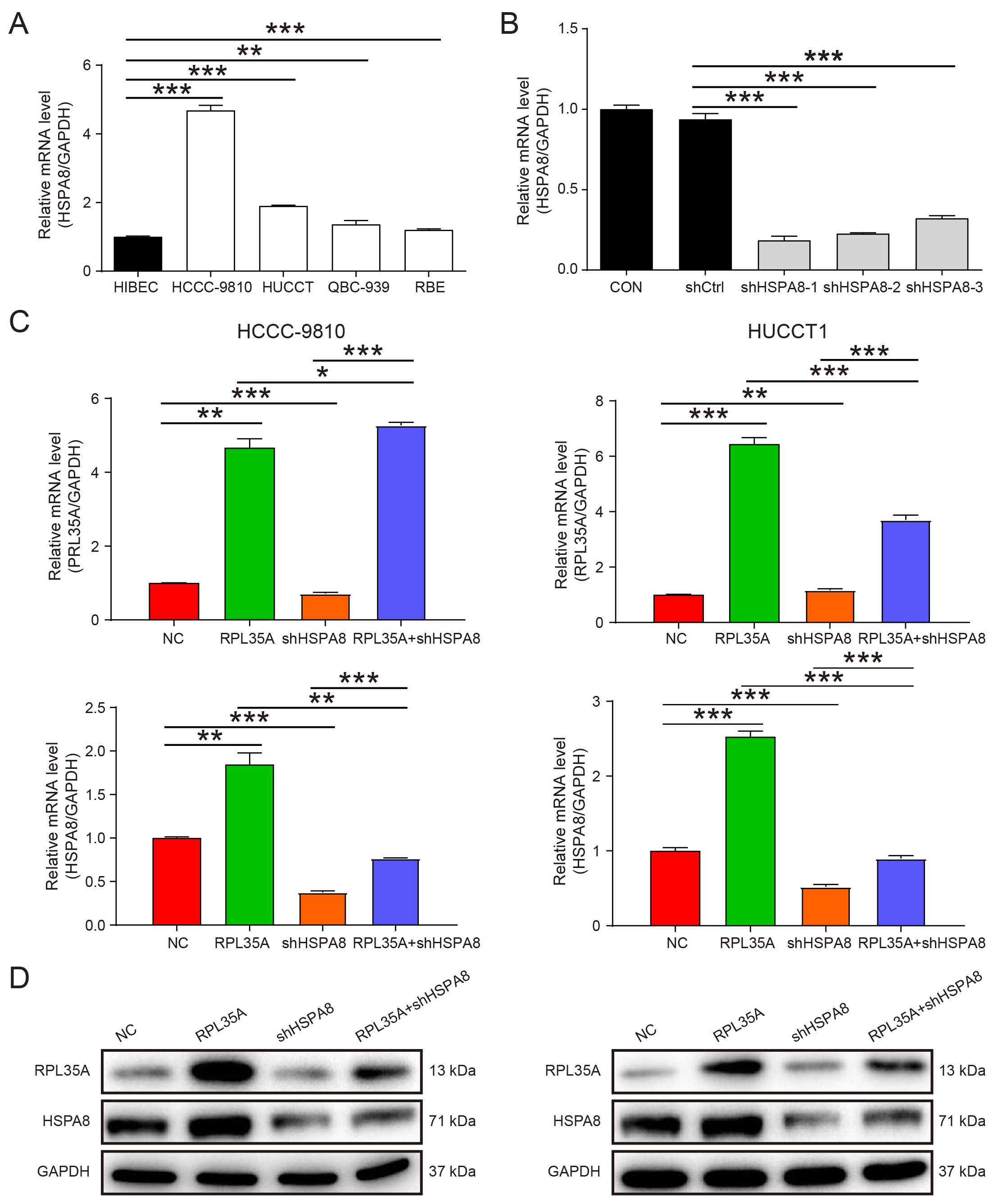


**Supplementary figure 3 Construction of RPL35A overexpression or/and HSPA8 knockdown CCA cells**

A. HSPA8 expression in 4 CCA cell lines (HCCC-9180, HUCCT1, QBC-939, and RBE) and human intrahepatic biliary epithelial cell line (HIBEC cells) was detected by RT-qPCR. B. RT-qPCR was used to detect the levels of HSPA8 mRNA in HCCC-9180 cells infected with three shHSPA8 lentiviruses. C. The expression of RPL35A and HSPA8 mRNA in HCCC-9180 and HUCCT1 cells of 4 groups was detected by RT-qPCR. D. The expression of RPL35A and HSPA8 protein in HCCC-9180 and HUCCT1 cells of 4 groups was detected by western blot. **P* < 0.05, ***P* < 0.01, ****P* < 0.001.


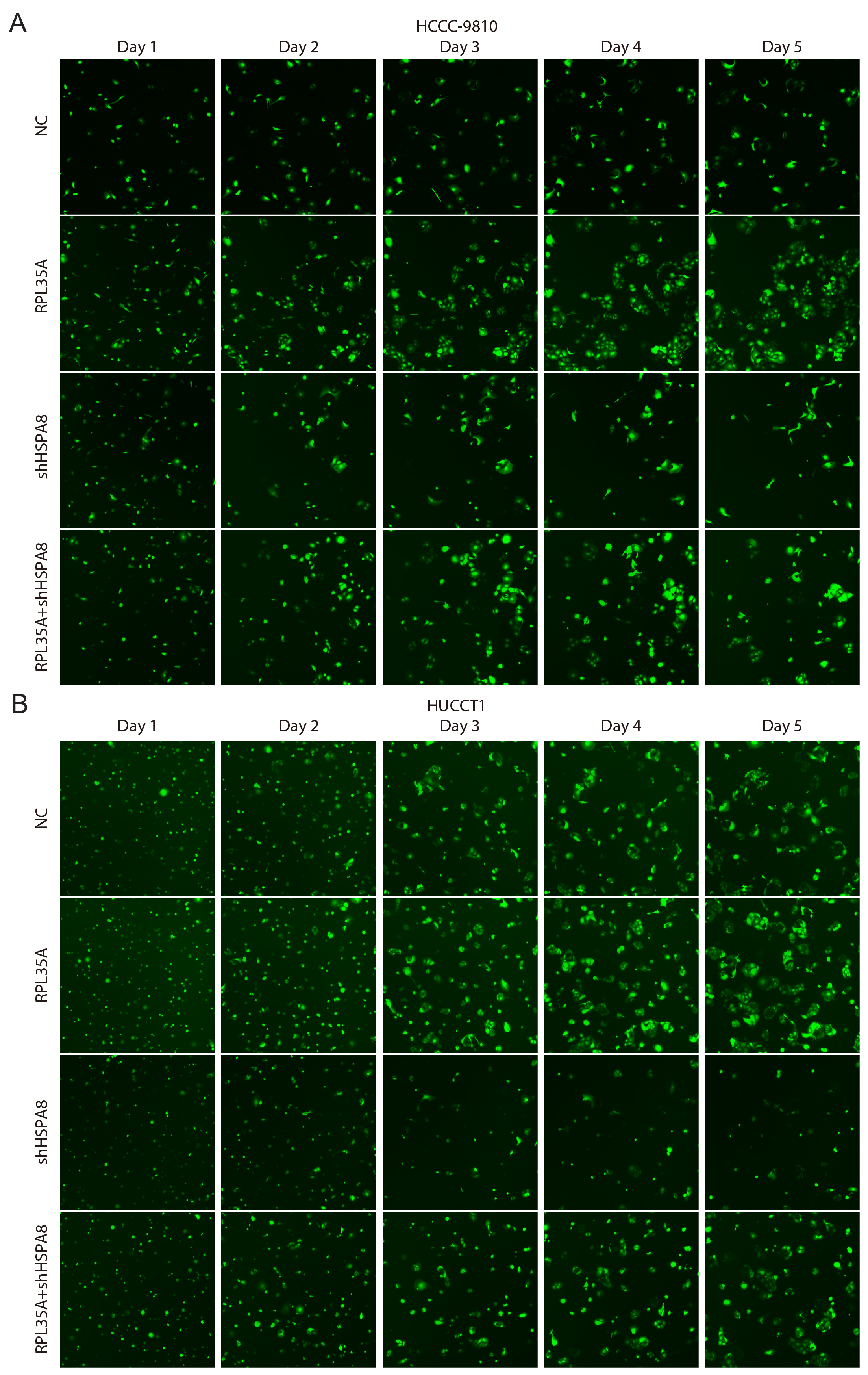


**Supplementary figure 4 Representative images of CCA cell proliferation assessed by Celigo cell count assay**

A. These were representative images of HCCC-9180 cells in each group detected by Celigo cell count assay. B. These were representative images of HUCCT1 cells in each group detected by Celigo cell count assay.

**Supplementary table 1.** Antibodies used in western blot, IHC and Co-IP

| Primary antibodies | Dilution in WB/Co-IP | Source species | Company | Catalog No. |
| --- | --- | --- | --- | --- |
| RPL35A | 1:2000 | Rabbit | abcam | Ab241070 |
| HSPA8 | 1:50/1:2000 | Rabbit | abcam | Ab51052 |
| Ubiquitin | 1:1000 | Mouse | Santa Cruz | sc-47721 |
| GAPDH | 1:3000 | Rabbit | Bioworld | AP0063 |
| Primary antibodies | Dilution in IHC | Source species | Company | Catalog No. |
| RPL35A | 1:100 | Rabbit | biorbyt | orb513214 |
| Ki67 | 1:100 | Rabbit | abcam | Ab16667 |
| Secondary antibody | Dilution |  | Company | Catalog No. |
| HRP Goat Anti-Rabbit IgG (WB and Co-IP) | 1:3000 |  | Beyotime | A0208 |
| HRP Goat Anti-Mouse IgG (WB and Co-IP) | 1:3000 |  | Beyotime | A0216 |
| HRP Goat Anti-Rabbit IgG (IHC) | 1:400 |  | Abcam | Ab97080 |

**Supplementary table 2.** Target sequences and shRNA sequences used for gene knockdown

| Gene symbol | Target sequence |  | shRNA sequences (5'-3') |
| --- | --- | --- | --- |
| RPL35A-1 | GGTGTTTACGCCCGAGATGAA | Pbr17813-a | ccggGGTGTTTACGCCCGAGATGAActcgagTTCATCTCGGGCGTAAACACCTTTTTG |
|  |  | Pbr17813-b | aattcaaaaaGGTGTTTACGCCCGAGATGAActcgagTTCATCTCGGGCGTAAACACC |
| RPL35A-2 | ACAGTCACTCCTGGCGGCAAA | Pbr17814-a | ccggACAGTCACTCCTGGCGGCAAActcgagTTTGCCGCCAGGAGTGACTGTTTTTTG |
|  |  | Pbr17814-b | aattcaaaaaACAGTCACTCCTGGCGGCAAActcgagTTTGCCGCCAGGAGTGACTGT |
| RPL35A-3 | TTGGACACAGAATCCGAGTGA | Pbr17815-a | ccggTTGGACACAGAATCCGAGTGActcgagTCACTCGGATTCTGTGTCCAATTTTTG |
|  |  | Pbr17815-b | aattcaaaaaTTGGACACAGAATCCGAGTGActcgagTCACTCGGATTCTGTGTCCAA |
